# Supplementary material for: Remapping in a recurrent neural network model of navigation and context inference
Source: bioRxiv. 2023 Jan 27:2023.01.25.525596. Preprint. [Version 2] doi: 10.1101/2023.01.25.525596 (PMC9900889; doi:10.1101/2023.01.25.525596)
Supplement: Supplementary file 1 [file NIHPP2023.01.25.525596v2-supplement-1.pdf]

bioRxiv preprint doi: https://doi.org/10.1101/2023.01.25.525596; this version posted January 27, 2023. The copyright holder for this preprint (which was not certified by peer review) is the author/funder, who has granted bioRxiv a license to display the preprint in perpetuity. It is made available under aCC-BY 4.0 International license.

Supplementary Information for: Remapping in a recurrent neural network model of navigation and context inference

Isabel I.C. Low,[1, 2,] * Lisa M. Giocomo,[3] Alex H. Williams[2, 4,] *

5. Zuckerman Mind Brain Behavior Institute, Columbia University
6. Center for Computational Neuroscience, Flatiron Institute
7. Department of Neurobiology, Stanford University
8. Center for Neural Science, New York University

* Correspondence to: il2419@columbia.edu, alex.h.williams@nyu.edu

bioRxiv preprint doi: https://doi.org/10.1101/2023.01.25.525596; this version posted January 27, 2023. The copyright holder for this preprint (which was not certified by peer review) is the author/funder, who has granted bioRxiv a license to display the preprint in perpetuity. It is made available under aCC-BY 4.0 International license.

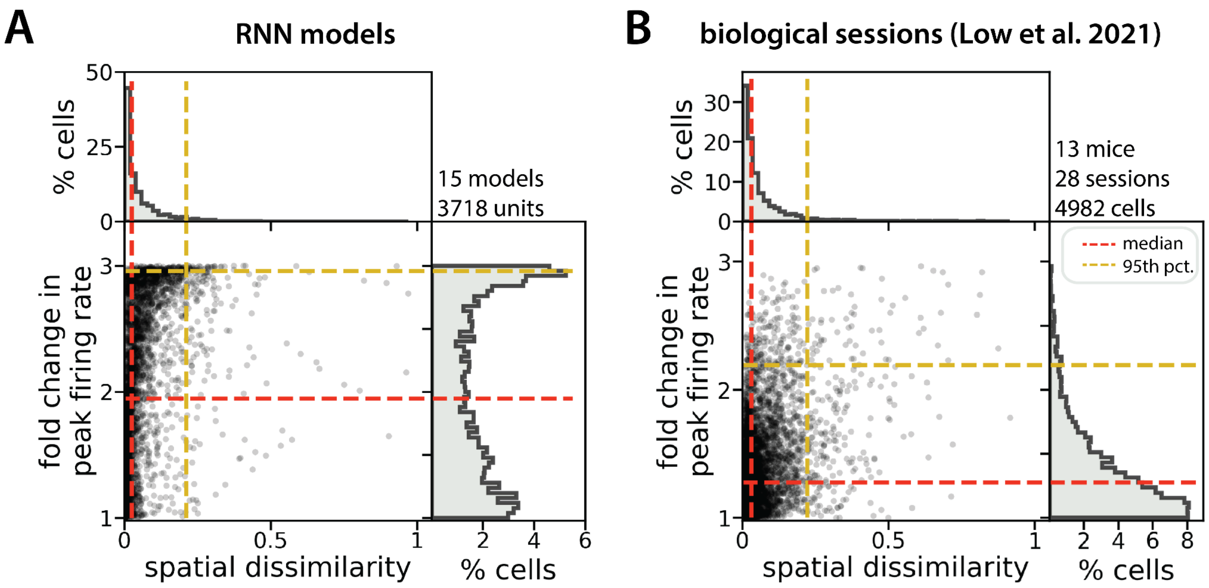

Supplemental Figure 1: single RNN units remap heterogeneously.
(A) Absolute fold change in peak firing rate versus spatial dissimilarity across latent state changes for all units from 2-map RNN models (points, single units; histograms, density distributions for each variable). Median change in peak firing rate (horizontal red dashes) = 1.9-fold; 95th percentile (horizontal gold dashes) = 3-fold. Median spatial dissimilarity (vertical red dashes) = 0.025; 95th percentile (vertical gold dashes) = 0.21.
(B) Modified from Low et al.[26] As in (A) but for biological neurons from 2-map sessions. Median change in peak firing rate (horizontal red dashes) = 1.28-fold. Median spatial dissimilarity (vertical red dashes) = 0.031; 95th percentile (vertical gold dashes) = 0.22.

bioRxiv preprint doi: https://doi.org/10.1101/2023.01.25.525596; this version posted January 27, 2023. The copyright holder for this preprint (which was not certified by peer review) is the author/funder, who has granted bioRxiv a license to display the preprint in perpetuity. It is made available under aCC-BY 4.0 International license.

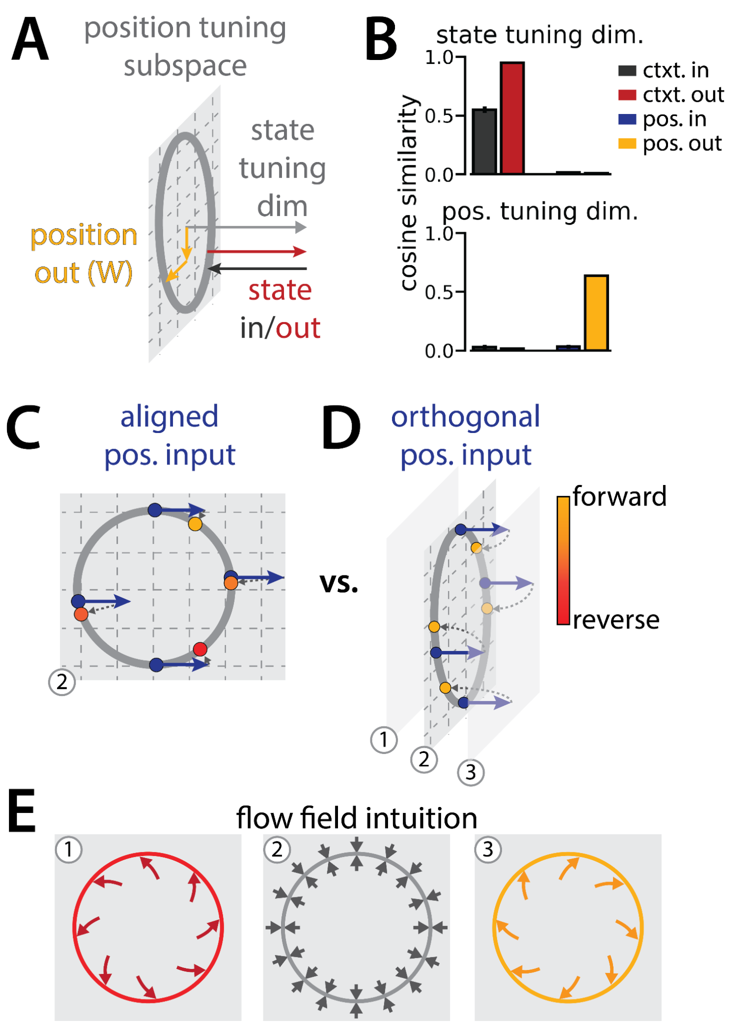

Supplemental Figure 2: RNN geometry is interpretable.
(A) Schematic showing the orthogonalization of the position and context input and output weights.
(B) Reproduced from Figure 1K.
(C-D) Schematic: How a single velocity input (blue arrows) updates the position estimate (yellow to red points) from the starting position (blue points).
(C) Velocity input lies in the position tuning subspace (gray plane)(hypothetical). Note that the same velocity input results in different final positions.
(D) Velocity input is orthogonal to the position tuning subspace (observed).
(E) Schematic of possible flow fields in each of the three planes (numbers correspond to planes in C and D), which would result in the correct positional estimate given orthogonal velocity inputs at different positions (D).

bioRxiv preprint doi: https://doi.org/10.1101/2023.01.25.525596; this version posted January 27, 2023. The copyright holder for this preprint (which was not certified by peer review) is the author/funder, who has granted bioRxiv a license to display the preprint in perpetuity. It is made available under aCC-BY 4.0 International license.

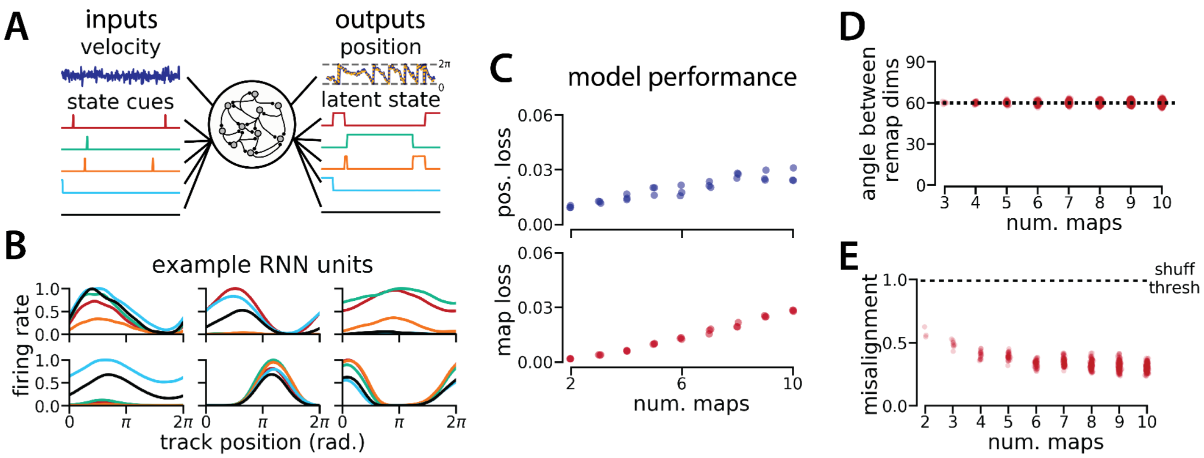

Supplemental Figure 3: Manifold geometry generalizes for up to 10 latent states.
(A) (Left) RNN models were trained to navigate in 1D (velocity signal, top) and discriminate between many (2–10) distinct contexts (transient, binary latent state signal, bottom). (Right) Example showing high prediction performance for position (top) and context (bottom) for 5 latent states.
(B) Position-binned activity for six example single RNN units, split by latent state (colors as in (A)).
(C) Models accurately estimated position (top) and latent state (bottom) for different numbers of latent states or "maps."
(D) Angle between remapping dimensions for different numbers of maps. The remapping dimensions were always maximally separated.
(E) Manifold misalignment score for all pairs of maps across all models (0, perfectly aligned; 1, p = 0.25 of shuffle). All pairs of manifolds were more aligned than expected by chance.

bioRxiv preprint doi: https://doi.org/10.1101/2023.01.25.525596; this version posted January 27, 2023. The copyright holder for this preprint (which was not certified by peer review) is the author/funder, who has granted bioRxiv a license to display the preprint in perpetuity. It is made available under aCC-BY 4.0 International license.

Supplemental Figure 4:

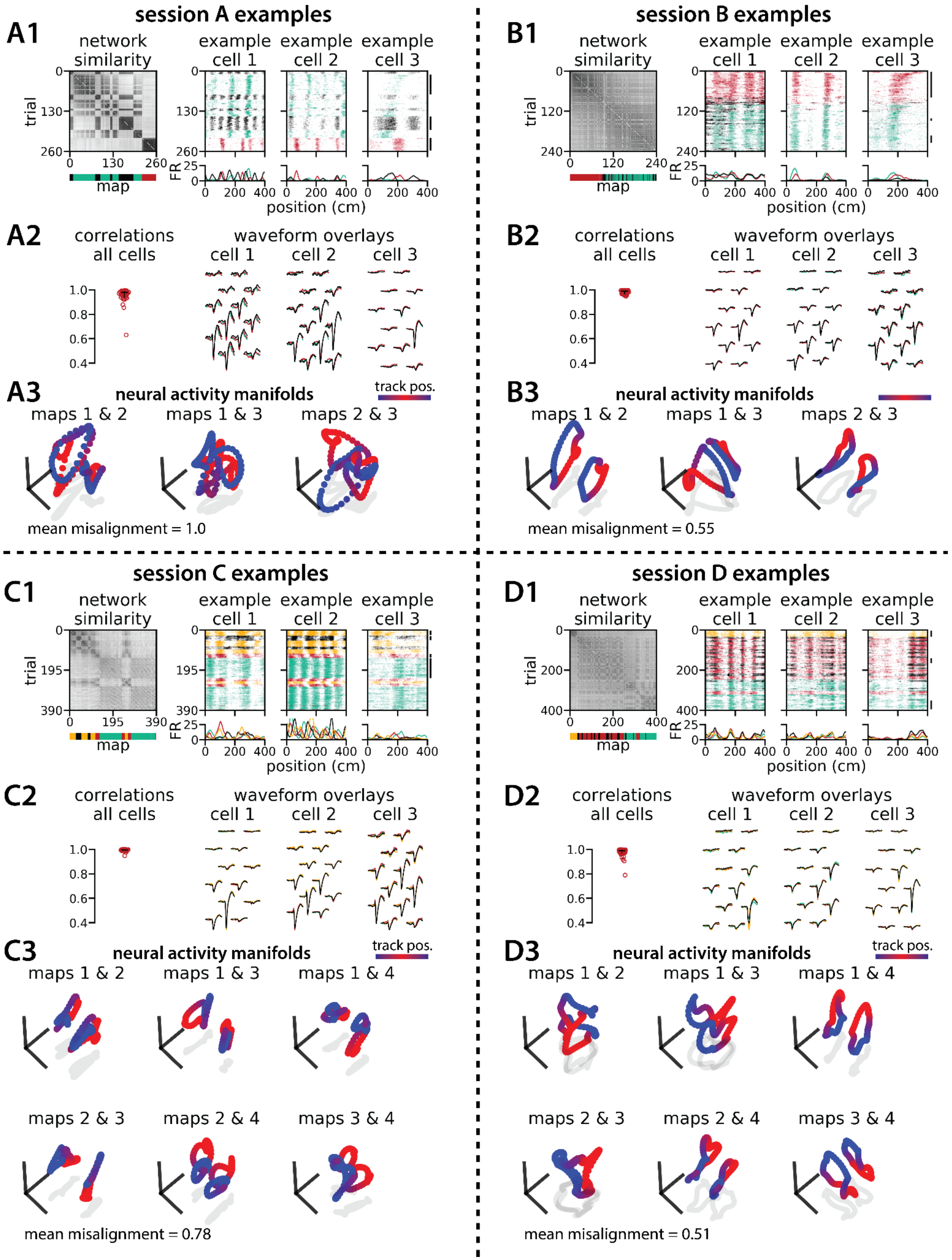

bioRxiv preprint doi: https://doi.org/10.1101/2023.01.25.525596; this version posted January 27, 2023. The copyright holder for this preprint (which was not certified by peer review) is the author/funder, who has granted bioRxiv a license to display the preprint in perpetuity. It is made available under aCC-BY 4.0 International license.

Supplemental Figure 4: Medial entorhinal cortex can remap between 3 or 4 maps and many geometric features are preserved.
Experimental data for 4 example multi-map sessions from Low et al.[26]
(A) Examples from the 3-map Session A (n = 142 cells).
(A1)(Left) Network-wide trial-by-trial correlations for the spatial firing patterns of all co-recorded neurons in the same example session (colorbar indicates correlation). (Left/bottom) k-means map assignments.
(Right) Three example medial entorhinal cortex neurons switch between three maps of the same track (top, raster; bottom, average firing rate by position; teal, map 1; red, map 2; black, map 3).
(A2) Comparisons of average spike waveforms sampled from each of the three maps (approximate sampling epochs indicated by black lines, A1 far right).
(Left) Across map correlations for average spike waveforms from all cells (horizontal bar, median = 0.978; vertical line, 5th – 95th percentile, 5th percentile = 0.935).
(Right) Overlay of average waveforms sampled from each map for the three example cells from (A2).
(A3) PCA projection of the manifolds associated with each pair of maps (colorbar indicates virtual track position). Text: average normalized manifold misalignment score for all pairs of maps (0, perfectly aligned; 1, p = 0.25 of shuffle).
(B–D) As in (A), but for the 3-map Session B (n = 184 cells; median waveform correlation = 0.988), 4-map Session C (n = 196 cells; median waveform correlation = 0.995), and 4-map Session D (n = 162 cells; median waveform correlation = 0.99).
